# The shared molecular mechanism of spinal cord injury and sarcopenia: a comprehensive genomics analysis

**DOI:** 10.3389/fneur.2024.1373605

**Published:** 2024-08-30

**Authors:** Binyang Wang, Xu Yang, Chuanxiong Li, Rongxing Yang, Tong Sun, Yong Yin

**Affiliations:** ^1^Department of Rehabilitation, The Affiliated Hospital of Yunnan University, Kunming, China; ^2^The Affiliated Hospital of Yunnan University, Kunming Medical University, Kunming, China

**Keywords:** spinal cord injury, sarcopenia, rehabilitation training, skeletal muscle, genomics

## Abstract

**Introduction:**

The occurrence of Spinal cord injury (SCI) brings economic burden and social burden to individuals, families and society, and the complications after SCI greatly affect the rehabilitation and treatment of patients in the later stage.This study focused on the potential biomarkers that co-exist in SCI and sarcopenia, with the expectation to diagnose and prognose patients in the acute phase and rehabilitation phase using comprehensive data analysis.

**Methods:**

The datasets used in this study were downloaded from Gene Expression Omnibus (GEO) database. Firstly, the datasets were analyzed with the “DEseq2” and “Limma” R package to identify differentially expressed genes (DEGs), which were then visualized using volcano plots. The SCI and sarcopenia DEGs that overlapped were used to construct a protein–protein interaction (PPI) network. Three algorithms were used to obtain a list of the top 10 hub genes. Next, validation of the hub genes was performed using three datasets. According to the results, the top hub genes were *DCN, FSTL1,* and *COL12A1,* which subsequently underwent were Gene Ontology and Kyoto Encyclopedia of Genes and Genomes enrichment analyses. We also assessed immune cell infiltration with the CIBERSORT algorithm to explore the immune cell landscape. The correlations between the hub genes and age and body mass index were investigated. To illustrate the biological mechanisms of the hub genes more clearly, a single-cell RNA-seq dataset was assessed to determine gene expression when muscle injury occurred. According to our analysis and the role in muscle, we chose the fibro/adipogenic progenitors (FAPs) cluster in the next step of the analysis. In the sub cluster analysis, we use the “Monocle” package to perform the trajectory analysis in different injury time points and different cell states.

**Results:**

A total of 144 overlapped genes were obtained from two datasets. Following PPI network analysis and validation, we finally identified three hub-genes (*DCN, FSTL1,* and *COL12A1*), which were significantly altered in sarcopenic SCI patients both before and after rehabilitation training. The three hub genes were also significantly expressed in the FAPs clusters. Furthermore, following injury, the expression of the hub genes changed with the time points, changing in FAPs cluster.

**Discussion:**

Our study provides comprehensive insights into how muscle changes after SCI are associated with sarcopenia by moving from RNA-seq to RNA-SEQ, including Immune infiltration landscape, pesudotime change and so on. The three hub genes identified in this study could be used to distinguish the sarcopenia state at the genomic level. Additionally, they may also play a prognostic role in evaluating the efficiency of rehabilitation training.

## 1 Introduction

Both traumatic and non-traumatic spinal cord injury (SCI) associated with unfavorable consequences. According to data from American National Spinal Cord Injury Database, the proportion of SCI patients in the United States is 0.0054%. In addition, the age of patients with SCI is gradually getting younger, which deserves our attention (1). A study by a Chinese team showed that the number of SCI patients in China is increasing at a rate of approximately 10% per year, and will gradually comprise a relatively large population in the future (2). Morever, the latest hospital-based retrospective study from China, it shows that the number of people with spinal cord injury between 35 and 45 approximately equal to the number of people over 65 years old, and most people with spinal cord injury are over 45 years old (3). So, there was an aging trend in spinal cord injury patients, and the older population was the most common population for sarcopenia (4). Again, It is vital to focusing on these SCI patients with sarcopenia. SCI can have serious effects on physiology, psychology, and family. There is a strong correlation between the cost of medical treatment after SCI and the level of tetraplegia; the cost of a high level of tetraplegia is the highest regarding both the initial hospitalization and subsequent treatment plan compared to other levels of tetraplegia (1).

When SCI occurs, the motor system, central nervous system, including the sensory system with its proprioceptive component, as well as the sympathetic nervous system, and other related systems, will all exhibit varying degrees of change. Preventing the deterioration of these conditions is one of the core treatments of SCI. The restoration of spinal cord function and remodeling of neural pathways is one of the research hotspots at present; however, there are still many challenges from application and transformation to implementation (5). Therefore, recovery after SCI also remains a concern. This is particularly true in patients with SCI at the neck level and thoracic level, who will experience a longer rehabilitation process after SCI and face a more severe rehabilitation challenge. Among these sequelae, skeletal muscle is a major concern. After SCI, the paralyzed muscles have reduced metabolism and motor capability (6). This feature has been reported in many studies, in addition to skeletal muscle capability changes from different perspectives, including from the aspects of a decline in quality, performance, strength (7–9).

Sarcopenia is characterized by progressive and widespread skeletal muscle dysfunction with adverse consequences (10). Sarcopenia can be diagnosed with several tests, which include the grip strength test, assessment of the appendicular skeletal muscle mass by dual-energy X-ray absorptiometry, the timed-and-go test, and other methods (10). As mentioned above, patients with SCI can present with a decrease in muscle mass, strength, and performance; however, we still lack the awareness to recognize, prevent, and change it. One study on patients with SCI with an average age of 38 years found that sarcopenia was present in many of them (11). Another study also found that sarcopenia and sarcopenic obesity were common in patients with SCI (12). However, at present, unique criteria and biomarkers for the early sensitive identification and specific diagnosis of SCI are lacking, which not only affects the life and quality of life of patients, but also results in a poor prognosis and complications (11, 13).

Research on the molecular mechanisms of the association between sarcopenia and SCI remains sparse. Studying the molecular relationship between the two diseases could result in early diagnosis, later treatment, and rehabilitation of these patients. Furthermore, it also meets the requirements for precision medicine. The aim of this study was to explore the co-pathogenesis between sarcopenia and SCI. We identified hub genes associated with the two diseases and uses various bioinformatics methods to explore the biological mechanisms. The hub genes identified for sarcopenia and SCI in this study are expected to provide new insights and ideas for the diagnosis, treatment, and rehabilitation of both diseases.

## 2 Materials and methods

The framework of this work shown in Supplementary Figure S1, and all steps will clearly illustrate in subsequent sections.

### 2.1 Collecting datasets

We obtained our datasets from Gene Expression Omnibus (GEO): (1) GSE21497 (14) contains data on 20 SCI patients; (2) GSE111016 (15) contains data on 20 healthy subjects and 20 sarcopenia patients; (3) GSE111010 (15) contains data on 14 healthy subjects, nine sarcopenia patients, five low muscle mass patients, and 11 low muscle strength or function patients; (4) GSE111006 (15) contains data on 28 healthy subjects, four sarcopenia patients, five low muscle mass patients, and three low muscle strength or function patients; (5) GSE117525 (16) contains data on 53 young subjects, 73 healthy older subjects, and 61 frail older subjects; (6) GSE142426 (17) contains data on 15 healthy subjects and 15 SCI patients; and (7) GSE138826 (18) contains single-cell transcriptomics data on seven SCI patients. We have listed the detailed information of the datasets in Table 1.

**Table 1:** Information on the datasets.

**Type**	**GEO number**	**Status**	**Tissue type**	**Organism**	**Sample number**
Microarray	GSE21497	Spinal Cord injury	Skeletal Muscle	Homo sapien	20
Bulk RNA-seq	GSE111016	Sarcopenia	Skeletal Muscle	Homo sapien	40
Bulk RNA-seq	GSE111010	Sarcopenia	Skeletal Muscle	Homo sapien	39
Bulk RNA-seq	GSE111006	Sarcopenia	Skeletal Muscle	Homo sapien	40
Microarray	GSE117525	Exercise Training	Skeletal Muscle	Homo sapien	259
Microarray	GSE142426	Spinal Cord injury	Skeletal Muscle	Homo sapien	30
Single-cell RNA-seq	GSE138826	Muscle Injury	Skeletal Muscle	*Mus musculus*	7

### 2.2 Identification of differentially expressed genes (DEGs)

We processed the GSE21497 dataset using the “Limma” (19) to identify the DEGs and draw the volcano plot. Notably, we merged the datasets (GSE111016, GSE111006, and GSE111010), and used the “sva” (20) package to remove the batch effects and draw the volcano plots. In this merged dataset, we also compared the low mass group and low muscle function group, as its provided phenotype information. In the analysis, we set the threshold at logFC (log fold change) ≥1 and *p* value <0.05 to distinguish the significant DEGs by “DESeq2” (21) package.

### 2.3 Gene Ontology (GO) and Kyoto Encyclopedia of Genes and Genomes (KEGG) enrichment analyses

To further explore the function behind DEGs, we used GO and KEGG enrichment analyses to investigate their biological significance using the “clusterProfiler” (22) package. In the GO analysis, we assessed the functional enrichment in three aspects, molecular function (MF), biological process (BP), and cellular component (CC). The *p* value thresholds of the GO and KEGG enrichment analyses were set as 0.01.

### 2.4 Protein–protein interaction (PPI) network construction

Firstly, we used the “venn” (23) package to obtain the genes that intersected in the GSE21497 and the merged datasets. Using the intersecting genes, we constructed the PPI network based on the STRING database (24). “Cytoscape” (Version 3.6) (25) was used to visualize the network. Finally, we used the “CytoHubba” (26) plugin to calculate the top genes based on the maximum neighborhood component algorithms, maximal correlation coefficient algorithms, and density of maximum neighborhood component algorithms.

### 2.5 Validation and evaluation of hub genes

We choose GSE117525, GSE21497, and GSE142426 as the validation datasets to verify the expression of hub genes. It is worth mentioning that GSE117525 and GSE14246 were both used to compare changes before and after rehabilitation training.

To provide the clinic value, we calculated the receiver operating characteristic (ROC) curves and area under the curve (AUC) with 95% confidence intervals (CI) using the “pROC” (27) and “plotROC” (28) packages to estimate the predictive accuracy of the hub genes. We the AUC to >0.60 to identify optimal biomarkers.

### 2.6 Immune cell infiltration landscape

We choose the GSE21497 and GSE111016 datasets to visualize the 28 types of immune cells by using gene expression using the CIBERSORT algorithm (29). A boxplot was used to exhibit the composition of immune cells in the datasets. We also used Spearman correlation analysis to evaluate the correlation between hub gene expression and immune cells between the different groups.

### 2.7 GO and KEGG enrichment analyses of hub genes

Based on the above analysis, we performed single gene analysis of the top hub genes. We also used the “clusterProfiler” package to conduct GO and KEGG enrichment analyses for the three genes to explore their underlying biological functions.

### 2.8 Correlation between body mass index (BMI) and age and hub genes

We further investigated the correlations between clinic information and hub genes using the “ggplot2” (30) package for the GSE21497 and GSE111016 datasets.

### 2.9 Single-cell data download and processing

The single-cell dataset GSE138826 was download from the GEO database. We filtered out the mitochondrial genes (15%) and hemoglobin genes (0.1%), identified cells with a total number of genes >300, and stipulated that genes must be expressed at least in three cells. All samples were eligible for further analysis.

We perform the SCTranform method to correct the batch effect using the “Seurat” (31) package. After finding the highly variable genes and scale data, the “RunPCA” package was used to reduce the dimension of the processed data. We choose dim = 15 to find cell clusters. Then, we used “UMAP” to perform the cell cluster visualization. We have listed our annotation genes for each cluster in Table 2. Furthermore, we used “Dotplot” and “Featureplot” to assess the expression level of each annotated gene to accurately mark these clusters.

**Table 2:** The top 10 hub genes by three different algorithms.

MNC			MCC			DMNC		
Rank	Name	Score	Rank	Name	Score	Rank	Name	Score
1	FBN1	7	1	FBN1	7	1	FBN1	7
1	DCN	7	1	DCN	7	1	DCN	7
3	COL12A1	5	3	COL12A1	5	3	COL12A1	5
3	COL6A3	5	3	COL6A3	5	3	COL6A3	5
5	FSTL1	4	5	FSTL1	4	5	FSTL1	4
6	GRIK2	3	6	GRIK2	3	6	GRIK2	3
6	GRIA1	3	6	GRIA1	3	6	GRIA1	3
6	MFAP5	3	6	MFAP5	3	6	MFAP5	3
6	ADAMTS5	3	6	ADAMTS5	3	6	ADAMTS5	3
10	KCNA1	2	10	KCNA1	2	10	KCNA1	2

### 2.10 Sub-cluster extraction and analysis

After visualizing the hub genes using “FeaturePlot,” a highly expressed subgroup (fibro/adipogenic progenitors (FAPs) cluster) of hub gene was extracted. After extracting the desired subpopulation of cells, we performed SCTranform and re-scaling of the raw counts, followed by dimensionality reduction and visualization using “UMAP.” In the FAPs, we listed our annotation genes for each cluster in Supplementary Table S1. The boxplot shows the expression difference in hub genes between different experimental time sequences.

We use “Monocle” (32) (Version 2) to perform the pseudo time trajectory analysis and identified the hub genes in the trajectory inference. The “Monocle” algorithm constructs the branches, and different branches represent cells in different trajectories.

## 3 Results

### 3.1 DEG identification

**Figure 1:**
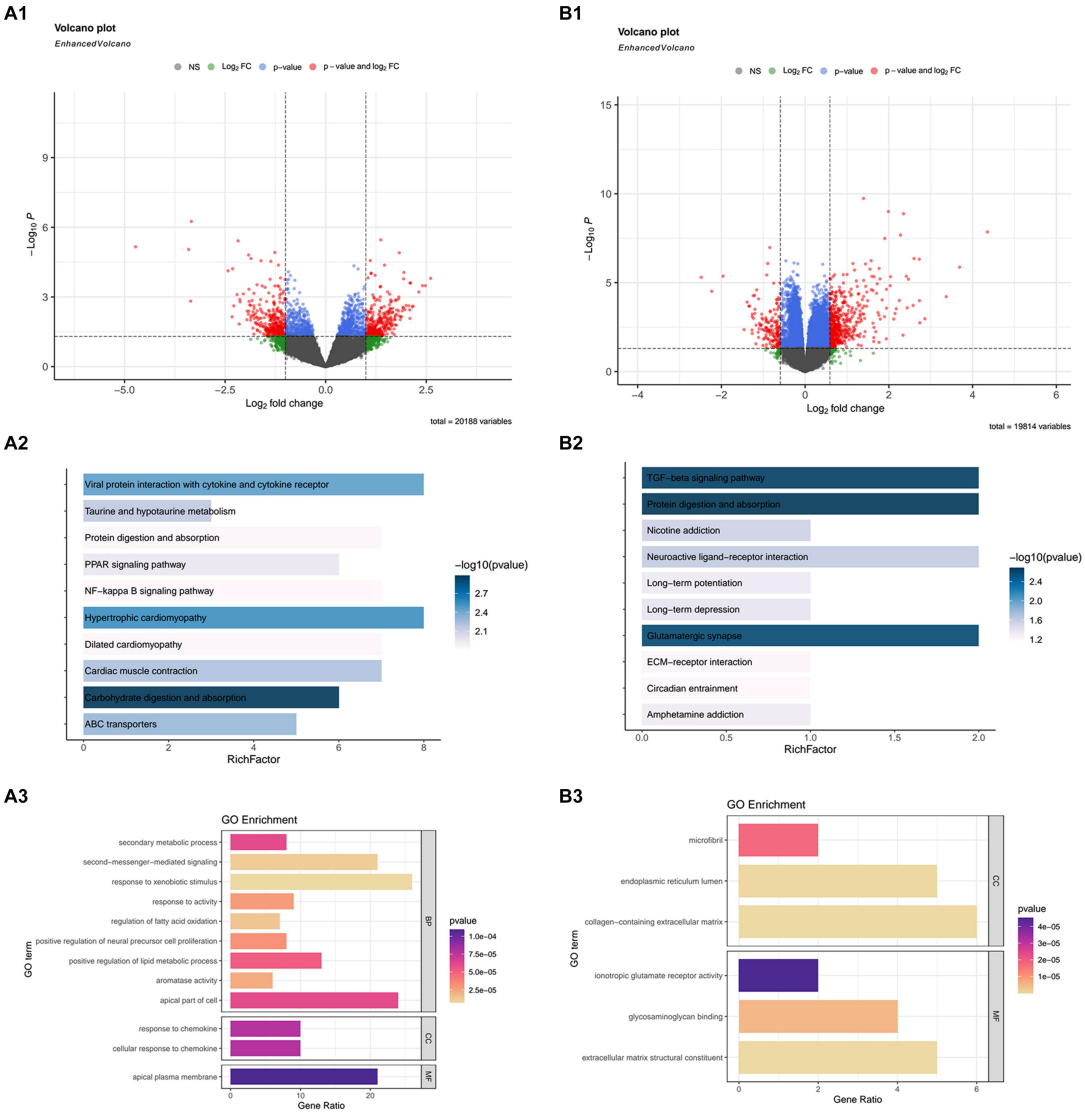
The differentially expressed genes (DEGs), and Kyoto Encyclopedia or Genes and Genomes (KEGG) and Gene Ontology (GO) enrichment analyses in GSE21497 and the merged datasets (including: GSE111016, GSE111010, and GSE111006). **(A1,B1)** Volcano map of DEGs in the GSE21497 and the merged dataset. **(A2,A3)** KEGG and GO enrichment analyses in GSE21497. **(B2,B3)** KEGG and GO enrichment analyses in the merged dataset.

In the GSE21497, we obtain 639 DEGs using the “Limma” package. Next, we merged three datasets, GSE111016, GSE111006, and GSE111010, and used the “DESeq2” package (Figure 1A1). In this merged data, after comparing healthy subjects and sarcopenia subjects, we obtained 7,032 DEGs (Figure 1B1).

### 3.2 Functional enrichment analysis

We conducted GO and KEGG analyses on the DEGs obtained from the above datasets, respectively, to discover the biological mechanism more clearly behind their respective DEGs.

Due to the excessive results obtained in the enrichment process, we selected the first few pathways with the smallest *p* value for display. In the GSE21497 dataset, the first few KEGG enrichment results were: carbohydrate digestion and absorption, hypertrophic cardiomyopathy, and viral protein interaction with cytokine and cytokine receptors. The top GO enrichment results were: apical plasma membrane, response to chemokines, and cellular response to chemokines (Figures 1A2,A3). Next, in the merged dataset (GSE11016, GSE111010, and GSE111006), the top KEGG enrichment results were: TGF-beta signaling pathway, protein digestion and absorption, and glutamatergic synapse. The top GO enrichment results were cytokine receptor binding, glycosaminoglycan binding, and receptor ligand activity (Figures 1B2,B3).

### 3.3 PPI network and identification of hub genes

**Figure 2:**
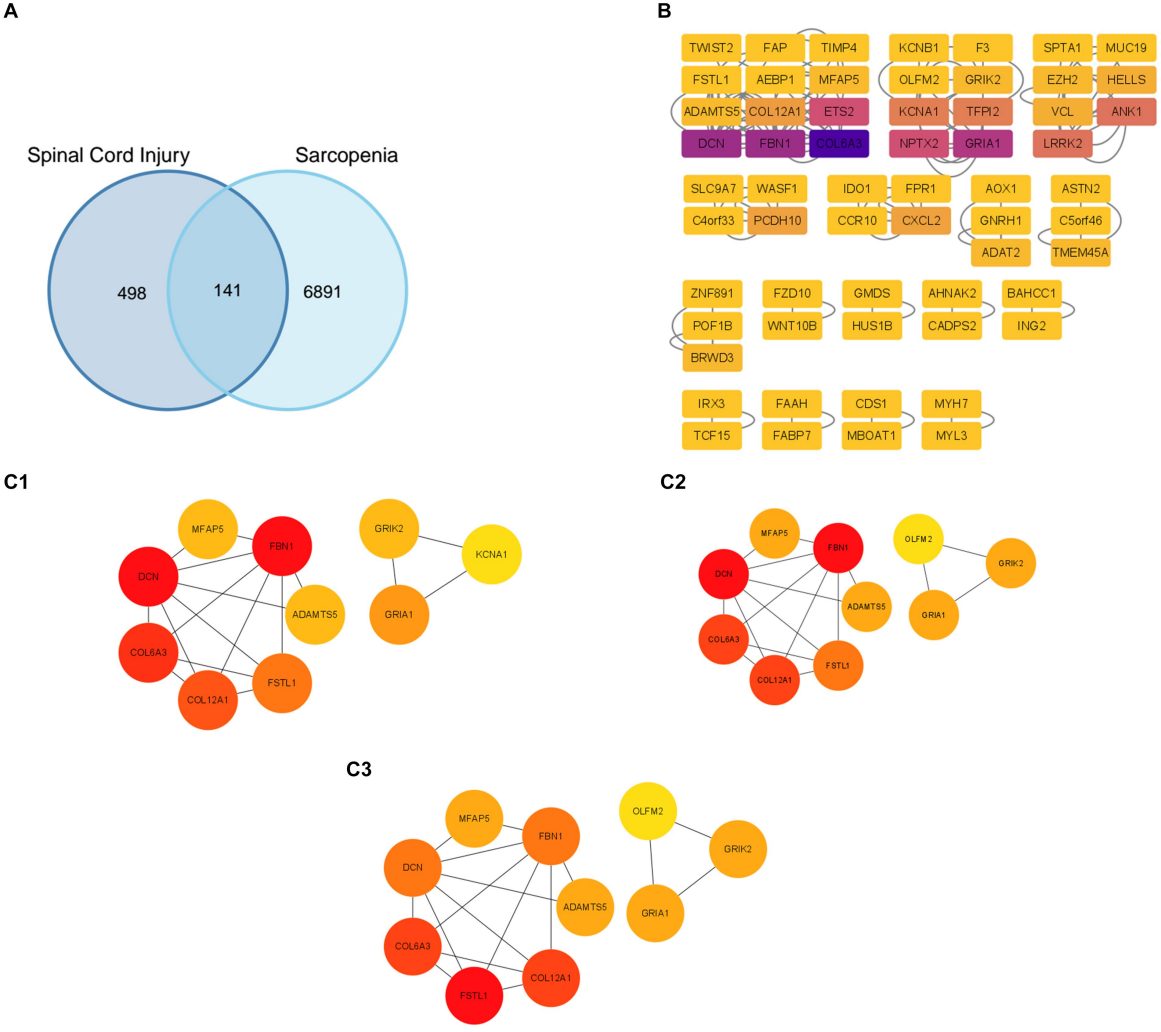
Protein–protein interaction (PPI) network construction and identification of hub genes. **(A)** The overlapped genes of the GSE21497 and the merged dataset. **(B)** The PPI network based on the STRING database. **(C1–C3)** Three different algorithms were used to obtain the hub genes, which included the MCC algorithm, MNC algorithm, and DMC algorithm.

Firstly, we used a Venn diagram to visualize the overlapping DEGs between the GSE21497 and merged dataset (Figure 2A); 141 DEGs overlapped. The STRING tool was used to construct the PPI network using the overlapping DEGs (Figure 2B). The PPI network contained 60 nodes and 116 edges; we set the interaction score at medium confidence. The “cytoHubba” plugin was applied in the PPI networks with the MNC algorithm, MCC algorithm, and DMNC algorithm (Figures 2C1–C3). Finally, 10 hub genes were selected for further analyses: *DCN, FBN1, COL6A3, FSTL1, COL12A1, ADAMTS5, MFAP5, TMP4, GRIA1,* and *GRIK2* (Supplementary Table S1).

### 3.4 Validation of hub genes

**Figure 3:**
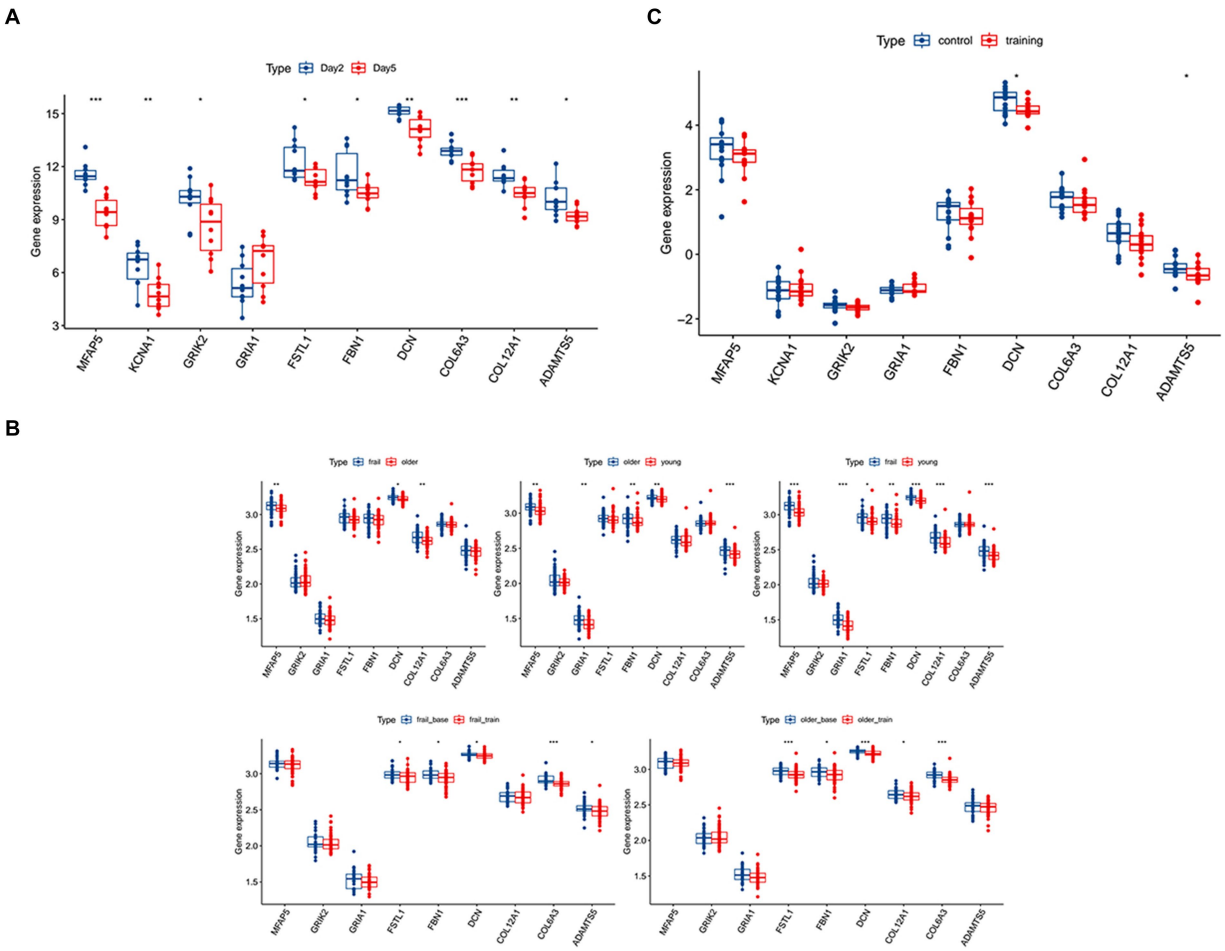
Validation of the hub genes and the diagnostic value of the hub genes in the three datasets. **(A)** The significance of the expression level of the three hub genes in GSE21497 between day 2 and day 5. **(B)** The significance of the expression level of the three hub genes in GSE142426 between the control group and training group in patients with spinal cord injury (SCI). **(C)** The significance of the expression level of the three hub genes in sarcopenia in the GSE117525 dataset. *p < 0.01, **p < 0.05, ***p < 0.001.

In the validation using the GSE117525 dataset, we compared the different training stage and different subjects’ statues to validate the hub genes (Figure 3C). In young vs. frail subjects and older vs. frail subjects, the expression of *DCN* remained significantly upregulated. The expression of *COL12A1* significantly changed in the comparison of frail vs. older subjects and frail vs. young subjects. After training, the expression of *DCN, FBN1, FSTL1*, and *COL6A3* were significant altered in both the frail subjects and older subjects’ groups. In the hub genes related to low muscle mass, the expression of *TGFB1* and *DCN* changed significantly after training in both frail subjects and older subjects.

In the validation using the GSE142426 dataset, we compared the SCI patients with healthy subjects after rehabilitation training in the lower limbs (Figure 3B). The change in expression of *DCN* and *ADAMTS5* were observed. In the hub genes related to low muscle mass, the expression *MSTN* also changed significantly.

In the validation using the GSE21497 dataset, we compared the SCI patients at different times (Figure 3A). We observed that on day 5 there was changes in the expression of several hub genes, including *DCN, FSTL1, COL12A1*, MFAP5, KCNA1, GRIK2, FBN1, COL6A3, ADAMTS5.

### 3.5 Evaluation of the diagnostic value of the hub genes

After validation with the above datasets, we selected *DCN, FSTL1*, and *COL12A1* for subsequent analyses and further evaluation. We evaluated the three hub genes against SCI and sarcopenia, respectively, using ROC curve analysis.

**Figure 4:**
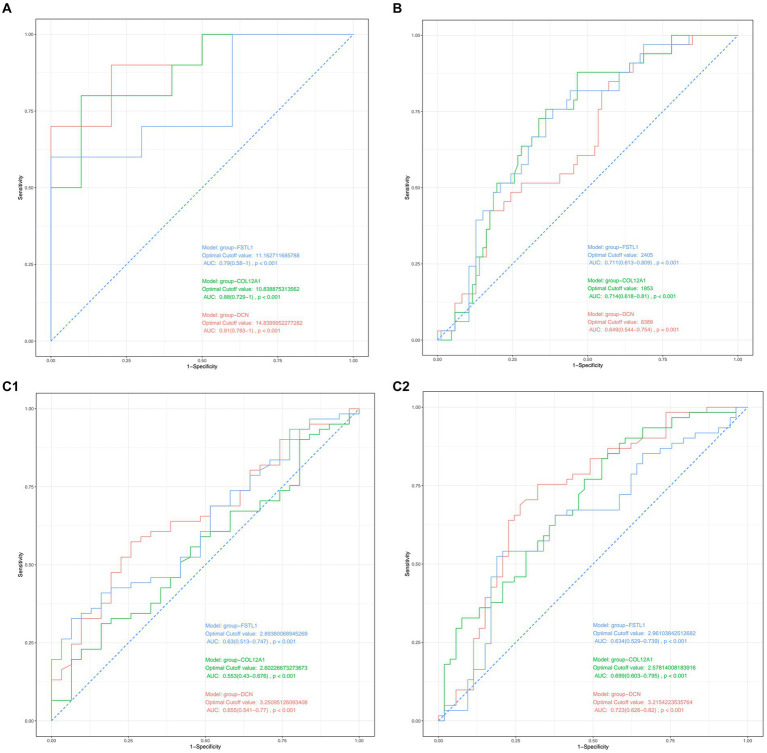
**(A)** Diagnostic value of the three hub genes in GSE21497. **(B)** Diagnostic values of the three hub genes in GSE142426. **(C1,C2)** Diagnostic value of the three hub genes in GSE117252.

In the GSE21497 and GSE142426 datasets, the AUC values for the three hub genes were > 0.60 (Figures 4A,B). In the GSE117525 dataset, we performed different comparisons to show the diagnosis value. In the group of frail subjects and young subjects, *DCN* and *FSTL1* showed AUC values >0.60; the AUC values of *COL12A1* were lower, but it still >0.55 (Figure 4C1). When comparing before and after training exercise, the AUC values of three the hub genes were > 0.60 (Figure 4C2).

### 3.6 The immune infiltration landscape of the hub genes

**Figure 5:**
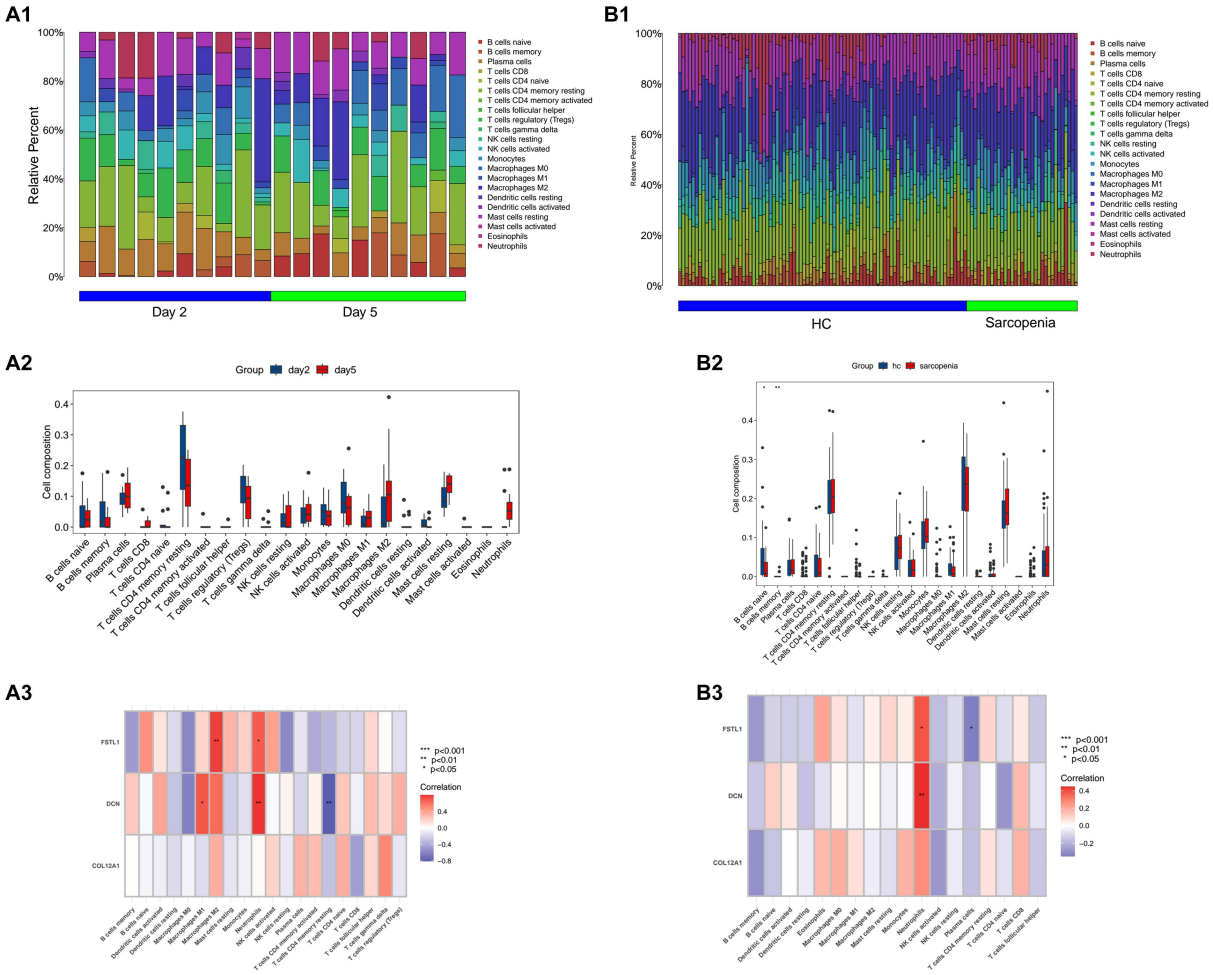
Immune infiltration and correlation with immune cells. **(A1,B1)** The composition and proportion of immune cells in the GSE21497 and the merged dataset. **(B2,B3)** The landscape of 28 immune cells in the GSE21497 and the merged dataset. **(A2,A3)** The correlation of the three hub genes with immune cells in the GSE21497 and the merged dataset (HC: healthy control).

The results of the CIBERSORT algorithm revealed the immune cell infiltration landscape in both the SCI and sarcopenia after comparing the different groups. In the GSE21497 dataset, we assessed the proportion of immune cells and landscape (Figures 5A1,A2). *FSTL1* had a significant correlation with M2 macrophages and neutrophils; *DCN* had a significant correlation with M1 macrophages, neutrophils, and CD4+ T cells (Figure 5A3). In the merged dataset (Figures 5B1,B2), *FSTL1* had a significant correlation with neutrophils and plasma cells; *DCN* had a significant correlation with neutrophils (Figure 5B3).

### 3.7 Enrichment analysis of the hub genes

**Figure 6:**
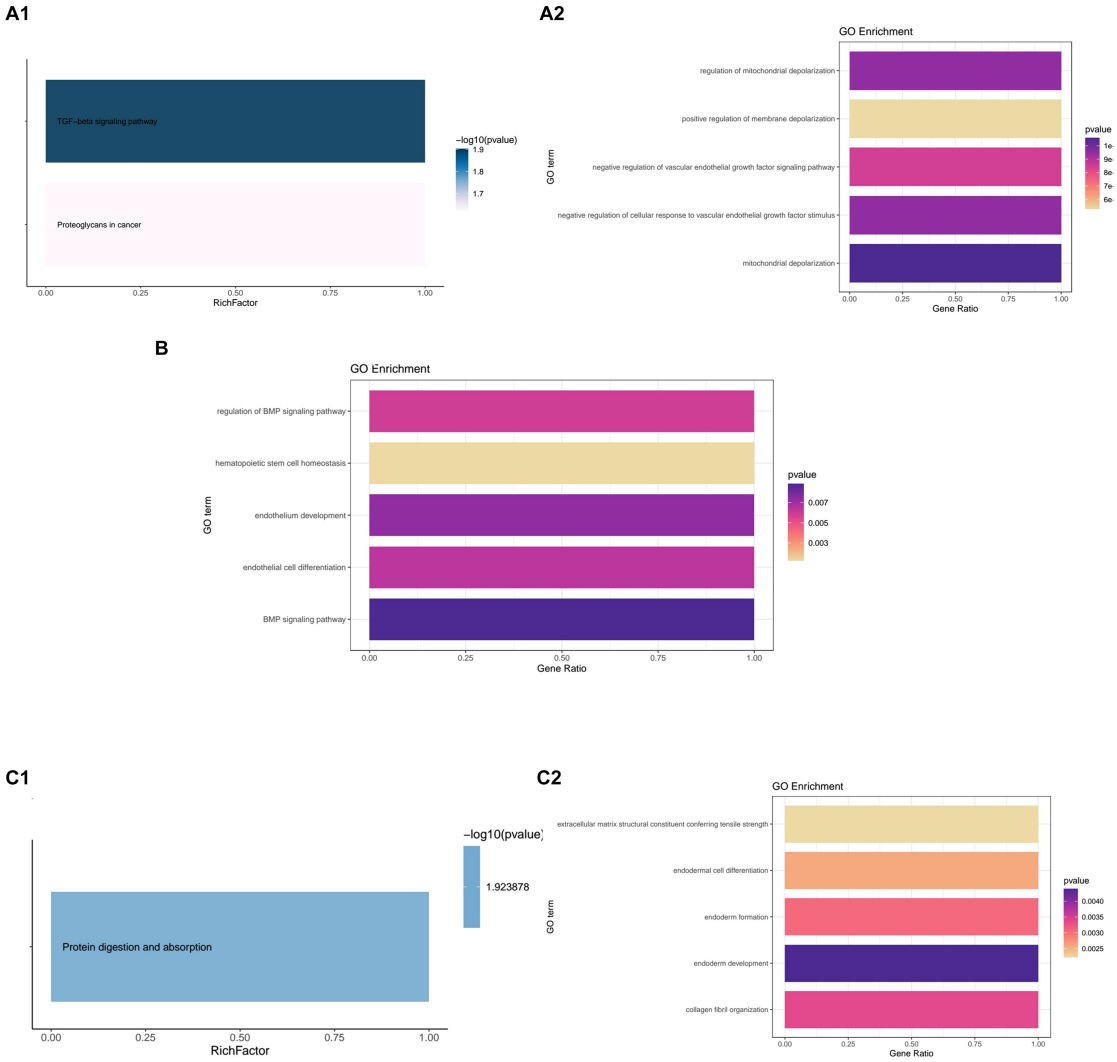
Kyoto Encyclopedia or Genes and Genomes (KEGG) and Gene Ontology (GO) enrichment of the hub genes. **(A1,A2)** KEGG and GO enrichment of *DCN*. **(B)** GO enrichment of *FSTL1*. **(C1,C2)** KEGG and GO enrichment of *COL12A1*.

*DCN* was markedly enriched in the TGF-beta signaling pathway in the KEGG analysis, and showed significant relationships with mitochondrial depolarization, regulation of mitochondrial depolarization, and negative regulation of cellular response to vascular endothelial growth factor stimulus. In the GO enrichment analysis, *DCN* was related to mitochondrial depolarization, regulation of mitochondrial depolarization, and negative regulation of cellular response to vascular endothelial growth factor stimulus (Figures 6A1,A2).

*FSTL1* failed to show enrichment in the KEGG analysis. However, in the GO analysis, it showed significant relationships with the BMP signaling pathway, endothelium development, and other relevant biological processes (Figure 6B).

In the KEGG enrichment, *COL12A1* only showed enrichment in protein digestion and absorption. In the GO enrichment, it showed strong relationships with endoderm development, collagen fibril organization, and endoderm formation (Figures 6C1,C2).

### 3.8 Correlations of hub genes with clinical features

**Figure 7:**
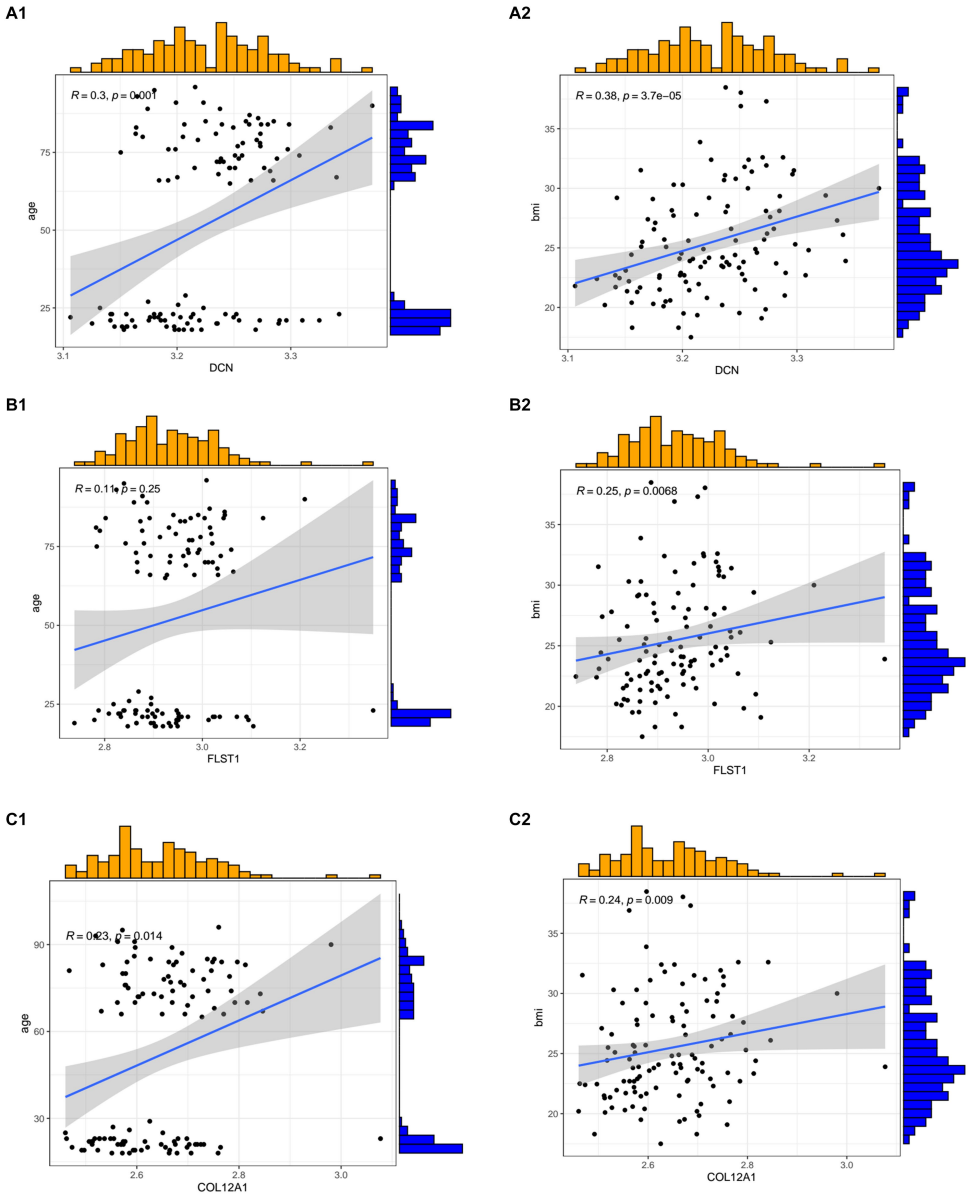
The relationships between the hub genes and age and body mass index. **(A1,A2)** The correlation between *DCN* and age and BMI. **(B1,B2)** The correlation between *FSTL1* and age and BMI. **(C1,C2)** The correlation between *COL12A1* and age and BMI.

In the GSE117525 dataset, we analyzed the relationships between age and BMI and hub genes. All three hub genes showed positive correlations with BMI and age (Figure 7).

### 3.9 Analysis of the single-cell RNA-seq dataset

**Figure 8:**
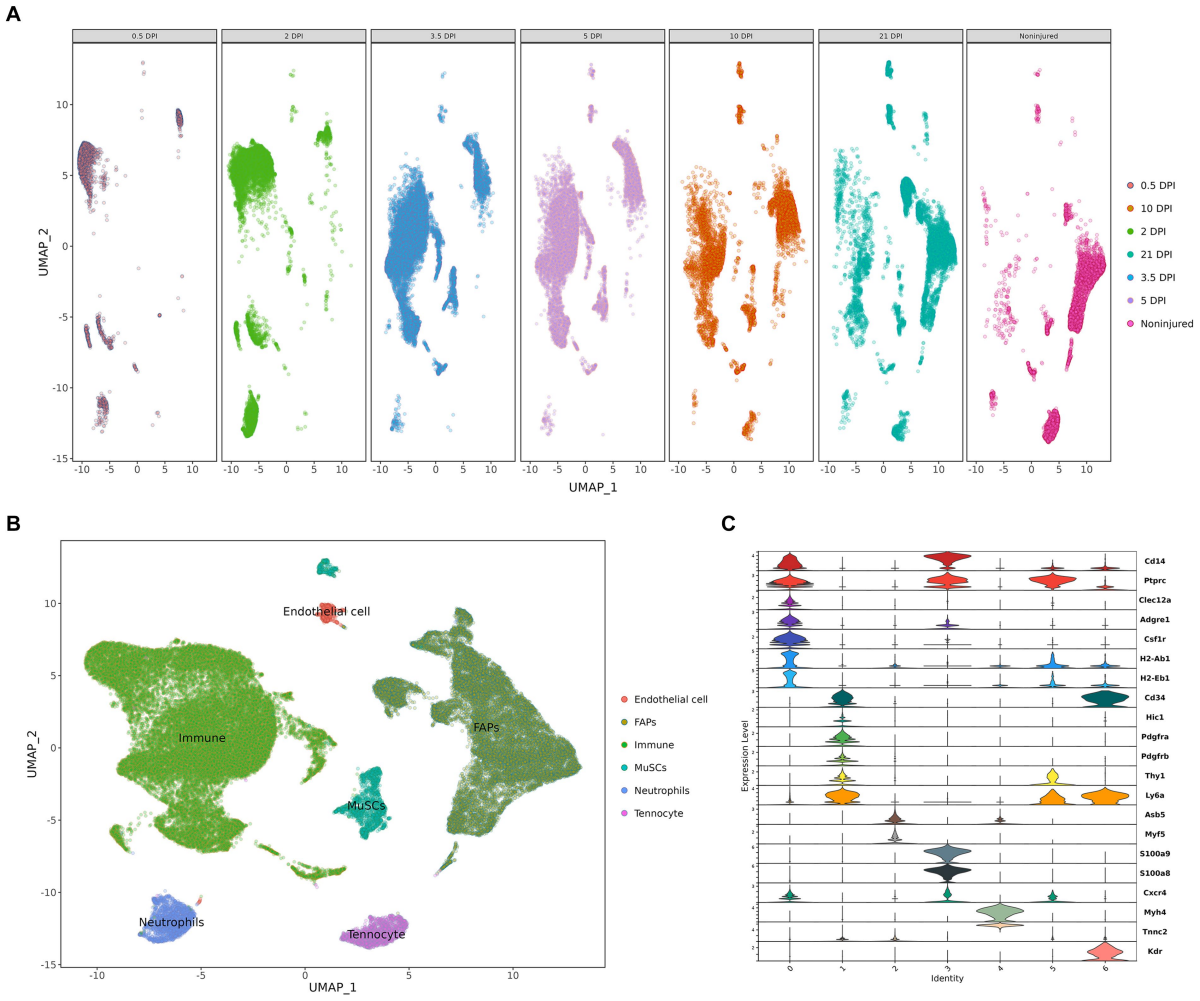
Cell clusters landscape and expression level. **(A)** The change in cell populations at different time points. **(B)** The UMAP visualization. **(C)** The violine plot show the expression level of annotation markers (DPI: days post injury).

After quality control, we obtained eight cell clusters, which were annotated with cell markers: *CD14, PTPRC, CLEC12A, ADGRE1, CSF1R, H2-AB1, H2-EB1, CD34, HIC1, PDGFRA, PDGFRB, THY1, LY6A, ASB5, MYF5, S100A8, S100A9, CXCR4, MYH4, TNNC2,* and *KDR* (Figure 8C). Finally, whole cells were allocated into eight cell clusters, including immune cells, FAP cells, MuSC cells, neutrophils, tenocytes, and endothelial Cells (Figure 8B). We also show the changes in cell clusters at different time points (Figure 8A).

**Figure 9:**
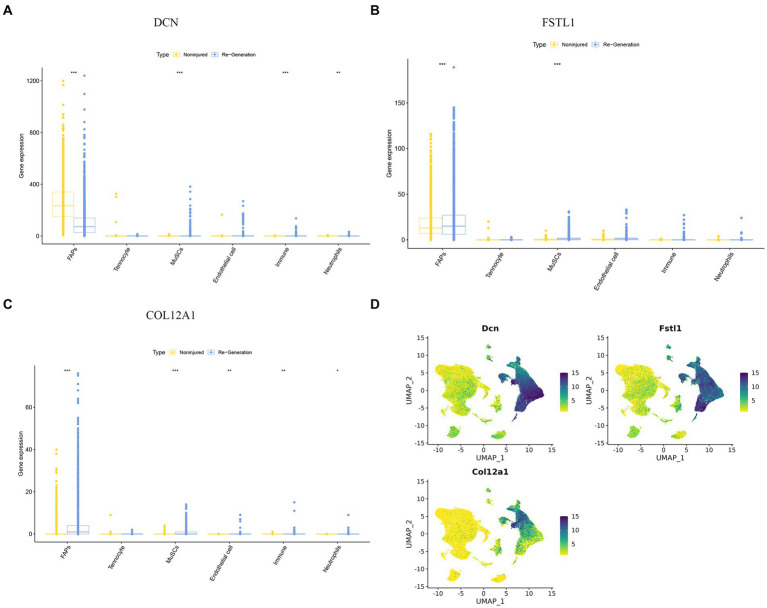
The expression difference of hub genes in different cell clusters. **(A–C)** A boxplot was used to visualize the expression difference of hub genes in difference cell clusters. **(D)** “Featureplot” was used to visualize the expression difference of the hub genes in difference cell clusters. *p < 0.01, **p < 0.05, ***p < 0.001.

The cell expression level of the three hub genes can be visualized in the boxplot, which clearly demonstrates that the FAPs cluster expression is significant (Figures 9A–C). We also show the expression level of the three hub genes using “featureplot” (Figure 9D). Moreover, FAPs play a crucial role in maintaining muscle homeostasis and promoting regeneration (33, 34).

### 3.10 Pseudotime trajectory analysis of the FAPs cluster

**Figure 10:**
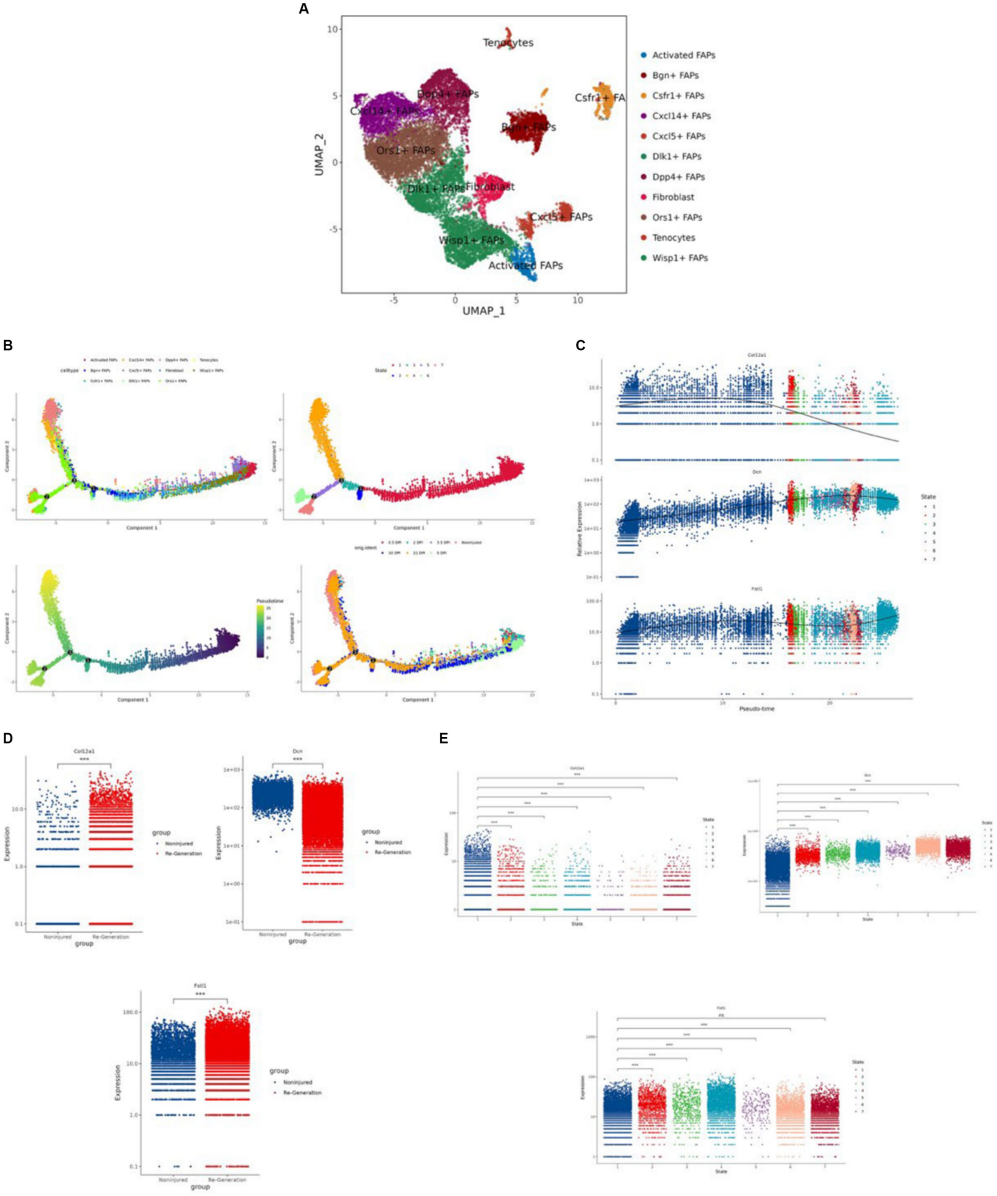
Identification of the fibro/adipogenic progenitors (FAPs) cluster and the regeneration trajectory-related analysis. **(A)** “UMAP” was used to visualize in the FAPs cluster. **(B)** The different trajectories: (1) trajectory inference analysis of FAPs by annotated markers; (2) trajectory inference analysis by different cell states; (3) psuedo time analysis colored by different shades of color; and (4) trajectory inference analysis by different time points. **(C)** The expression difference of the hub genes in different cells in trajectory interference. **(D)** The expression difference of the hub genes in the different groups. **(E)** The expression level of the hub genes in different cell states. *p < 0.01, **p < 0.05, ***p < 0.001.

After extracting the FAPs cluster, we obtained the 11 clusters and annotated them, including Ors1+ FAPs, Wisp1+ FAPs, Dlk1+ FAPs, Dpp4+ FAPs, Cxcl14+ FAPs, Bgn + FAPs, fibroblasts, Cxcl5+ FAPs, Csfr1+ FAPs, activated FAPs, and tenocytes (Figure 10A).

We used “Monocle” to reduce the dimension with the “UMAP” method and to construct the pseudotime trajectory. After calculating the score of the pseudotime value, we obtained the pseudotime trajectory. Firstly, we performed the trajectory analysis according to the 11 cell types (Figure 10B). Through the evaluation of different states, we obtained seven differentiation states; red is the earliest differentiation state, and to the left is the subsequent differentiation state (Figure 10B). From the trajectory, we can also see that the darker the blue, the earlier the differentiation, and the lighter the blue, the later the differentiation (Figure 10B). Furthermore we show the pseudotime trajectory at different time points (Figure 10B).

According to the expression level of the three hub genes, we assessed the modulation change patterns. The expression of *COL12A1* showed a decreasing trend. The expression of *DCN* and *FSTL1* showed the same ascending trend, although the former one had a greater change rate. Furthermore, both peak at the end of trajectory (Figure 10C).

We also compared the expression level of the hub genes by group and differentiation states (Figures 10D,E).

## 4 Discussion

The correlation between SCI and sarcopenia has gained increasing attention in recent years. Following SCI, skeletal muscle atrophy is approximately 30–60%, and changes, such as decreased muscle endurance and increased fatigability, often occur (35). Patients with SCI are more likely to develop sarcopenia than the general population due to limb dysfunction, especially those with high spinal segmental injury, long-term bed state, or reduced nutritional intake (11, 13, 36, 37). In the 2019 European consensus, the criteria for sarcopenia included low muscle strength, low muscle quantity or quality, and low physical performance (10). In recent years, people have also become increasingly aware of the emergence of sarcopenia after SCI and have implemented many rehabilitation measures to improve muscle changes, such as electrical stimulation, exercise training, and other rehabilitation therapies (7, 38).

After the acute phase of treatment ends for many patients with SCI, most need to enter the rehabilitation phase. This link is extremely important for patients, not only to improve the remaining function, but also to replace the lost function learning. When a SCI occurs, muscle mass decreases significantly after 6 weeks; however, muscle mass can be increased by body weight supported treadmill training (BWSTT) or functional electrical stimulation (FES) (39). Low muscle quality and strength also decrease after SCI, and patients with a high injury plane showed a greater decrease (40, 41). Infiltration of intramuscular fat is also associated with muscle loss; however, FES and resistance training can improve these two symptoms to a certain extent (41). In addition to exercise training, the use of some drugs, such as testosterone and B2-adrenergic agonists, have been shown to improve muscle loss (42). In addition, in patients with SCI, the vastus lateralis show higher fibrosis compared with healthy controls, and muscle training, such as resistance training, could reduce muscle fibrosis (43, 44). However, there is still a lack of research on the effects of training at the genetic level.

Normal muscle tissue has a degree of regeneration that resists muscle loss, but beyond this level (especially after disease or aging), this function does not compensate for loss (45). The regeneration of muscle tissue is a coordinated result of multiple cells, among which satellite cells, FAP cells, have shown important regeneration potential in recent years (46, 47). FAPs plays an important role in muscle homeostasis and regeneration (33, 34). In a muscle where no injury has occurred, FAPs are in a quiescent condition. Furthermore, they are the predominant mononuclear cell population and an essential mesenchymal progenitor for supporting function (48). After muscle injury, FAPs will wake related cell populations to participant in regeneration. In an animal model experiment, researchers used *Pdgfrα* knock-out mice to simulate depletion of skeletal muscle, which showed a muscle regeneration delay, significant regenerative deficit, and a decrease in the muscle index (49). Furthermore, in sarcopenia-related research, when the depletion of FAPs occurs, it causes a reduction in muscle strength and weight (50). This is why FAPs was selected as a sub-cell cluster to conduct further analysis in this study.

In our study, we focus on the mechanism of SCI and sarcopenia in the muscle tissue. After validation, we obtained three hub genes: *DCN, FSTL1*, and *COL12A1*. According to the clinic information supplied by the datasets, we performed a correlation analysis between the hub genes and clinical information. The result showed age and BMI had a positive relationship with the hub genes.

DCN is an avid collagen-binding protein and, and in many skeleton and muscle diseases, DCN plays different roles due to its role in the pericellular matrix and extracellular matrix (51–53). DCN has been widely shown to regulate cartilage degeneration, bone matrix in osteoporosis, tendon architecture, and functional activity (54–56). In contrast, research on the association of DCN in SCI and sarcopenia is lacking. In SCI, few researchers focus on the muscular changes; most assess the transformation of the central nervous system. Because DCN can suppress scarring effects and inhibit fibrogenesis, researchers want to use it to reverse the scarring response after nervous system injury (57, 58). As previously mentioned, skeletal muscle loss in SCI patients can result in fibrosis; however, consistent rehabilitation training can alleviate this effect. It could be assumed that DCN may work in this training process and suppress fibrosis. In out results, *DCN* showed significant changes after training. Furthermore, in the KEGG enrichment analysis of *DCN*, it was found to play a crucial role in the TGF-beta signaling pathway, which may promote fibrosis, and if the pathway is suppressed, it will prevent fibrosis and improve regeneration (59–61). When the expression of *DCN* is elevated, the effects on the TGF-beta signaling pathway decline. Furthermore, *DCN* also participates in process of muscle regeneration. According to the single-cell RNA-seq analysis, the expression of *DCN* showed a significantly difference in the FAPs cell population, with an increasing trend in pseudotime trajectory. In the GO enrichment, analysis the result showed that *DCN* is related to mitochondrial function. There are few studies to show a correlation between *DCN* and mitochondrial function in muscle tissue; however, mitochondrial function, including maximal mitochondrial ATP production, dysregulation of mitochondrial dynamics, and mitochondrial proteolysis and mitophagy, may affect the muscle mass, function, and quality (62). In the skeletal muscle of patients with SCI, a mitochondrial oxygenation capability deficit has been observed, which is related to components such as succinate dehydrogenase decrease; rehabilitation training could increase mitochondrial function (63).

FSTL1 is a secreted factor from skeletal muscle; it participates in revascularization during ischemia and is related to the function of angiogenesis in skeletal muscle (64). For the capability of normal tissue, blood supply is critical, which is determined by vascular formation (65). In muscle regeneration, when mesenchymal or other stem cell’s function, the decisive factor is whether there is existing vasculature (66). In addition, after exercise, *FSTL1* expression is elevate in the serum (67). However, evidence is lacking regarding a correlation between *FSTL1* and indicies of muscle, including mass, quality, and performance. In the GO enrichment analysis, *FSTL1* was shown to be related to the BMP signaling pathway, endothelial cell differentiation, and development. Research shows that BMP signaling controls muscle mass by the Smad family, and perturbed BMP signaling would lead to muscle loss (68, 69). Furthermore, BMP signaling relies on satellite cells to promote muscle regeneration. The links between *FSLT1*, BMP signaling, and satellite cells are elusive (70). There were no outcomes in the KEGG analysis of *FSTL1*; however, some studies have shown that it is related to Samd2/3 signaling and integrin β3/Wnt signaling (71, 72).

COL12A1 belongs to the family of fibril-associated collagens with interrupted triple helical domains and regulates cell communication, tissue repair, and regeneration (73, 74). *COL12A1* is widely expressed in mesenchymal tissues in the embryo, which is consistent with our results in the GO enrichment analysis (75). In *COL12A1*-related myopathic Ehlers–Danlos syndrome, patients usually present with global muscle weakness and atrophy (76). In the KEGG analysis, *COL12A1* was related to protein digestion and absorption. The skeletal muscle stores approximately 75% of the protein in the whole body, so when muscle loss or other sarcopenic situations begin, the balance between protein production and consumption is lost (77, 78). In patients with SCI, they may deplete more protein; therefore, it is vital for them to pay more attention to protein intake, and muscle loss is one of the recommendation standards for increasing protein intake (78). Thus, it is a worth exploring markers of skeletal muscle-related dysfunction.

In the immune infiltration landscape in SCI muscle tissue, there is no difference when comparing the time point of day 2 and day 5. We deem that the distance between the two time points is too short to make the difference significant. In sarcopenia datasets, compared with healthy control, the B cell naïve and B cell memory change significantly. Research shows that the capacity of B cells relates to skeletal regeneration and muscle strength (79). *DCN* and *FSTL1* significantly correlated with M1 and M2 macrophages. Myogenesis and myotube fusion correlate with M2-biase macrophages and M1 macrophages convert to M2 macrophages in skeletal muscle regeneration (80). Furthermore, the infiltration of neutrophils will lead to fibrosis in muscle fiber regeneration, which significantly correlates with *DCN* and *FSTL1* (79).

There are some limitations in our study should be acknowledged. Firstly, in microarray datasets or bulk-RNA seq datasets, patients with SCI are not formally diagnosed with sarcopenia. In that situation, we used the related sarcopenia datasets. Secondly, clinical information is lacking in many datasets, which made it hard for us to find the exact evidence and correlations between the hub genes and clinical features. Even although we still used limited clinical information to perform the correlation, a larger sample is needed to validate its value. In our future research, we will validate these hub genes *in vitro* and clearly explore the mechanism between SCI and sarcopenia in muscle tissue.

## 5 Conclusion

In our study, we identified three hub genes (*DCN, FSTL1*, and *COL12A1*) by bioinformatics analysis of GEO datasets. In patients with SCI, these hub genes show the underlying mechanism of the development of sarcopenia and could be a diagnostic and prognostic marker. We also provide the predictive values in a rehabilitation view to understand the clinical values of the hub genes, which will help in clinical decision-making. Furthermore, we present the immune change in SCI with sarcopenia. In general, the new sights provided by our study may promote the exploration of the comorbidity of SCI and sarcopenia. *DCN, FSTL1,* and *COL12A1* are new candidate biomarkers for the comorbidity of SCI and sarcopenia.

## Data Availability

Information for existing publicly accessible datasets is contained within the article.

## Supplementary material

SUPPLEMENTARY FIGURE S1 The workflow of work.
