# Supplementary material for: The shared molecular mechanism of spinal cord injury and sarcopenia: a comprehensive genomics analysis
Source: Front Neurol. 2024 Aug 30;15:1373605. doi: 10.3389/fneur.2024.1373605 (PMC11392746; doi:10.3389/fneur.2024.1373605)
Supplement: SUPPLEMENTARY FIGURE S1 — The workflow of work. [file Table_1.docx]

| **Type** | **GEO Number** | **Status** | **Tissue Type** | **Organism** | **Sample Number** |
| --- | --- | --- | --- | --- | --- |
| Microarray | GSE21497 | Spinal Cord injury | Skeletal Muscle | Homo sapien | 20 |
| Bulk RNA-seq | GSE111016 | Sarcopenia | Skeletal Muscle | Homo sapien | 40 |
| Bulk RNA-seq | GSE111010 | Sarcopenia | Skeletal Muscle | Homo sapien | 39 |
| Bulk RNA-seq | GSE111006 | Sarcopenia | Skeletal Muscle | Homo sapien | 40 |
| Microarray | GSE117525 | Exercise Training | Skeletal Muscle | Homo sapien | 259 |
| Microarray | GSE142426 | Spinal Cord injury | Skeletal Muscle | Homo sapien | 30 |
| Single-cell RNA-seq | GSE138826 | Muscle Injury | Skeletal Muscle | Mus musculus | 7 |
