# Supplementary material for: The shared molecular mechanism of spinal cord injury and sarcopenia: a comprehensive genomics analysis
Source: Front Neurol. 2024 Aug 30;15:1373605. doi: 10.3389/fneur.2024.1373605 (PMC11392746; doi:10.3389/fneur.2024.1373605)
Supplement: Supplementary file 2 [file Table_2.docx]

| **Cell Type** | **Annotation** |
| --- | --- |
| Immune Cell | Cd14,Ptrc,Clec12a,ADGRE1,Csf1r,H2-AB1,  H2-EB1 |
| FAPS | Cd34,Hic1,Pdgfra,Pdgfrb,Thy1,ly6a |
| MuSCs | Asb5,Myf5 |
| Neutrophils | S100a9,S100a8,Cxcr4 |
| Tennocyte | Myh4,Tnnc2 |
| Endothelial cell | Kdr |
