# Supplementary figures and images for: The shared molecular mechanism of spinal cord injury and sarcopenia: a comprehensive genomics analysis
Source: Front Neurol. 2024 Aug 30;15:1373605. doi: 10.3389/fneur.2024.1373605 (PMC11392746; doi:10.3389/fneur.2024.1373605)

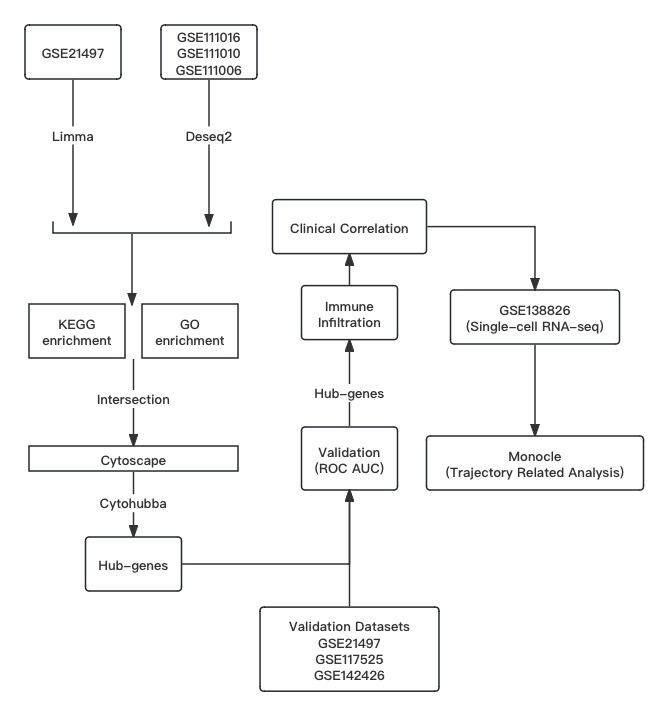

Supplement: Supplementary file 3 [file Image_1.jpeg]
